# Cost-effectiveness of everolimus-eluting versus bare-metal stents in ST-segment elevation myocardial infarction: An analysis from the EXAMINATION randomized controlled trial

**DOI:** 10.1371/journal.pone.0201985

**Published:** 2018-08-16

**Authors:** Nadine Schur, Salvatore Brugaletta, Angel Cequier, Andrés Iñiguez, Antonio Serra, Pilar Jiménez-Quevedo, Vicente Mainar, Gianluca Campo, Maurizio Tespili, Peter den Heijer, Armando Bethencourt, Nicolás Vazquez, Marco Valgimigli, Patrick W. Serruys, Zanfina Ademi, Matthias Schwenkglenks, Manel Sabaté

**Affiliations:** 1 European Center for Pharmaceutical Medicine (ECPM), University of Basel, Basek, Switzerland; 2 University Hospital Clínic, Institut d’Investigacions Biomèdiques August Pi i Sunyer (IDIBAPS), Barcelona, Spain; 3 University Hospital of Bellvitge, Barcelona, Spain; 4 Hospital do Meixoeiro, Vigo, Spain; 5 University Hospital of Sant Pau, Barcelona, Spain; 6 University Hospital San Carlos, Madrid, Spain; 7 Hospital General of Alicante, Alicante, Spain; 8 University Hospital Ferrara, Ferrara, Italy; 9 University Hospital Bolognini Seriate, Bergamo, Italy; 10 Amphia Ziekenhuis, Breda, Netherlands; 11 Hospital Son Espases, Palma de Mallorca, Spain; 12 Hospital Juan Canalejo, A Coruña, Spain; 13 Erasmus MC, Rotterdam, Netherlands; 14 University Hospital of Bern, Inselhospital, Bern, Switzerland; 15 International Centre of Circulatory Health, Imperial College London, London, United Kingdom; UMCU, NETHERLANDS

## Abstract

**Background:**

Use of everolimus-eluting stents (EES) has proven to be clinically effective and safe in patients with ST-segment elevation myocardial infarction but it remains unclear whether it is cost-effective compared to bare-metal stents (BMS) in the long-term. We sought to assess the cost-effectiveness of EES versus BMS based on the 5-year results of the EXAMINATION trial, from a Spanish health service perspective.

**Methods:**

Decision analysis of the use of EES versus BMS was based on the patient-level clinical outcome data of the EXAMINATION trial. The analysis adopted a lifelong time horizon, assuming that long-term survival was independent of the initial treatment strategy after the end of follow-up. Life-expectancy, health-state utility scores and unit costs were extracted from published literature and publicly available sources. Non-parametric bootstrapping was combined with probabilistic sensitivity analysis to co-assess the impact of patient-level variation and parameter uncertainty. The main outcomes were total costs and quality-adjusted life-years. The incremental cost-effectiveness ratio was expressed as cost per quality-adjusted life-years gained. Costs and effects were discounted at 3%.

**Results:**

The model predicted an average survival time in patients receiving EES and BMS of 10.52 and 10.38 undiscounted years, respectively. Over the life-long time horizon, the EES strategy was €430 more costly than BMS (€8,305 vs. €7,874), but went along with incremental gains of 0.10 quality-adjusted life-years. This resulted in an average incremental cost-effectiveness ratio over all simulations of €3,948 per quality-adjusted life-years gained and was below a willingness-to-pay threshold of €25,000 per quality-adjusted life-years gained in 86.9% of simulation runs.

**Conclusions:**

Despite higher total costs relative to BMS, EES appeared to be a cost-effective therapy for ST-segment elevation myocardial infarction patients due to their incremental effectiveness. Predicted incremental cost-effectiveness ratios were below generally acceptable threshold values.

## Introduction

Coronary artery disease and specifically ST-elevation myocardial infarction (STEMI) represent a leading cause of mortality and morbidity worldwide [1, 2]. Percutaneous coronary intervention (PCI) represents the gold standard treatment in STEMI and recent studies and meta-analyses have shown that the use of everolimus-eluting stents (EES) is safer and more effective than the use of bare metal stents (BMS). EES are associated with lower rates of stent thrombosis, myocardial infarction and cardiac mortality [3]. The EXAMINATION (clinical Evaluation of the Xience-V stent in Acute Myocardial INfArcTION) trial, in particular, compared the efficacy and safety of EES *versus* BMS in patients with STEMI requiring emergency PCI [4] and demonstrated a clinical superiority of durable polymer-based EES over cobalt chromium BMS, with a significant reduction of the combined patient-oriented endpoint of all-cause death, any myocardial infarction, or any revascularisation at 5-year follow-up [5].

Given strained healthcare budgets and the higher acquisition costs of EES over BMS, economic considerations need to complement the assessment of clinical effectiveness. It is still unclear whether in a STEMI clinical scenario, EES are also preferable over BMS in terms of long-term, incremental cost-effectiveness. Such information is essential for decision makers at the clinical and health policy levels.

The purpose of the present study was to evaluate the long-term cost-effectiveness of EES versus BMS from a Spanish health service perspective. The analysis was designed to assess incremental costs and quality-adjusted life years (QALYs) during a life-long time horizon, based on the 5-year results of the EXAMINATION trial.

## Materials and methods

We used the 5-year, patient-level clinical outcome data of the EXAMINATION trial. Within-trial analysis was combined with modelling elements to incorporate quality of life and costs, and to extend the analysis beyond the 5-year trial observation period. Required life-expectancy estimates, health-state utility scores and unit costs representing 2016 prices were extracted from published literature. Clinical effectiveness was assessed by translating the reduction in clinical event occurrence into estimates of clinical effectiveness expressed as QALYs gained. It was assumed that long-term survival is independent of the initial treatment strategy after the end of follow-up. The model evaluated and compared total costs and QALYs between the EES and BMS strategies. On this basis, the cost-effectiveness of EES versus BMS was expressed as the incremental cost-effectiveness ratio (ICER), which is the difference in total costs between the two treatments divided by the difference in QALYs gained. Costs and effects were discounted at 3%. Non-parametric bootstrapping was combined with probabilistic sensitivity analysis to co-assess the impact of patient-level variation and parameter uncertainty. Details are provided in the following sections.

### Design and results of the EXAMINATION trial

Details of the EXAMINATION trial have been reported elsewhere [4]. In short, EXAMINATION was a multicenter, multinational, prospective, randomized, two-arm, single-blind, controlled trial done in eight Spanish, two Italian and two Dutch sites. STEMI patients were eligible if they reported to the treating center up to 48 h after the onset of symptoms, required emergency PCI and had vessel sizes of 2.25 to 4.00 mm to allow for the implantation of stents. Exclusion criteria were pregnancy, STEMI secondary to stent thrombosis, chronic treatment with anti-vitamin K agents, and known intolerance to aspirin, clopidogrel, heparin, stainless steel, everolimus, or contrast material. All sites received the approval of their medical ethics committee and all patients provided written informed consent for participation in the trial. The local medical ethics committees were: Clinical Research Ethics Committee of the University Hospital Clínic de Barcelona (CEIC Hospital Clínic), Barcelona, Spain; Clinical Research Ethics Committee of the Ciutat Sanitària de Bellvitge (CEIC Bellvitge), Barcelona, Spain; Ethics Commission of the Hospital do Meixoeiro, Vigo, Spain; Ethics Commission of the University Hospital San Carlos, Madrid, Spain; Ethics Commission of the Hospital General of Alicante, Alicante, Spain; Ethics Commission of the University Hospital Ferrara, Ferrara, Italy; Ethics Commission of the Amphia Ziekenhuis, Breda, Netherlands; Ethics Committee of the Hospital Son Espases, Palma de Mallorca, Spain; and Ethics Commission of the Hospital Juan Canalejo, A Coruña, Spain.

Eligible patients were randomly assigned to receive either EES or BMS in a 1:1 ratio. In total, 1498 patients, 751 in the EES and 747 in the BMS group, were followed over a 5-year period or until death. Primary endpoint of the study was the patient-oriented, combined endpoint of all-cause death, any myocardial infarction, or any revascularization at 1 year as suggested by the Academic Research Consortium (ARC) [6]. Secondary endpoints included the device-oriented combined endpoint of cardiac death, target vessel myocardial infarction, or target lesion revascularization [6], as well as all-cause and cardiac death, any myocardial infarction (WHO extended definition [7]), target lesion revascularization, target vessel revascularization and stent thrombosis (according to ARC [6]). All of the above endpoints were assessed up to the end of the 5-year follow-up period. Detailed definitions of the endpoints are provided elsewhere [4]. A summary of the clinical event and endpoint rates at 5-year follow-up is provided in Table 1 [5].

**Table 1:** Clinical events at 5-year follow-up [5].

	EES group (n = 751)	BMS group (n = 747)	Hazard ratio (95% CI)	p value
Patient-oriented endpoint*	159 (21%)	192 (26%)	0.80 (0.65–0.98)	0.033
Device-oriented endpoint†	88 (12%)	113 (15%)	0.75 (0.57–0.99)	0.043
Death§	65 (9%)	88 (12%)	0.72 (0.52–1.00)	0.047
Cardiac	47 (6%)	55 (7%)	0.84 (0.57–1.24)	0.370
Vascular	4 (1%)	5 (1%)	0.79 (0.21–2.92)	0.720
Non-cardiovascular	14 (2%)	28 (4%)	0.49 (0.26–0.92)	0.027
Myocardial infarction	35 (5%)	27 (4%)	1.27 (0.77–2.10)	0.350
Target vessel related	21 (3%)	23 (3%)	0.90 (0.50–1.62)	0.710
Non-target vessel related	15 (2%)	6 (1%)	2.44 (0.95–6.29)	0.070
Revascularization	93 (12%)	116 (16%)	0.77 (0.59–1.01)	0.060
Target lesion	32 (4%)	54 (7%)	0.57 (0.37–0.89)	0.012
Target vessel	49 (7%)	76 (10%)	0.62 (0.43–0.89)	0.009
Non-target vessel	62 (8%)	62 (8%)	0.98 (0.69–1.39)	0.910
Definite or probable stent thrombosis	15 (2%)	23 (3%)	0.64 (0.33–1.23)	0.180

EES = everolimus-eluting stent. BMS = bare metal stent.

*Combined (hierarchical) endpoint of all-cause death, any recurrent myocardial infarction, and any revascularisation.

†Combined (hierarchical) endpoint of cardiac death, target vessel myocardial infarction, and target lesion revascularisation.

§In accordance with the Academic Research Consortium (ARC) recommendations [6].

The trial showed a reduction in the rate of the combined patient-oriented endpoint of EES over BMS. The benefit was mainly attributable to reduced all-cause death (9% vs. 12%), non-cardiac death (2% vs. 4%) and target-lesion revascularization rates (4% vs. 7%). It occurred immediately after implantation and also at long-term follow-up for up to five years. Very late hazards in the extended clinical follow-up, such as stent thrombosis or target vessel myocardial infarction, were been reported.

### Long-term survival and utilities

Long-term survival beyond five years of follow-up was based on the average life expectancy by age group using World Life Expectancy estimates [8] for Spain. It was reduced by estimates of the amount of years of potential life lost after acute MI, adjusted to fit the age range of the EXAMINATION trial population, as published by Bucholz et al. (2015) [9]. Consequently, cardiovascular events after five years were not explicitly modelled. Long-term survival was assumed to be independent of the initial treatment strategy for those who survived the 5-year follow-up period.

Health-state utility scores based on European Quality of Life-5 Dimensions (EQ-5D) population norms for Spain, using country-specific time trade-off values [10], were included in the model as a starting point. We then introduced decreases in utility due to the initial STEMI event, repeat MI and STT events, revascularization, PCI and CABG procedures. EQ-5D population norms[10] reductions were additionally applied to incorporate the effect of ageing on utility scores. Health status changes in the first year and subsequent years after the initial MI were taken into account using estimates published by Lacey and Walters [11] which were adjusted to the mean age of the EXAMINATION trial population. Further MI or stent thrombosis events during the observational period were considered to affect the health-state only half as much as the initial event. It was assumed that the full impact of a repeat event lasts for a year and that a reduced impact occurred in the subsequent years. Revascularization, CABG and/or PCI were considered to have the same impact on utility as repeat MI or STT events, for a year. However no effect was assumed for subsequent years. Utility values and decrements used are presented in Table 2.

**Table 2:** Model inputs for probabilities, utilities and costs.

Parameter	Base case value	Range of variation	Basis of variation	References
**Unit costs (in 2016 €)**				
Stent				
Everolimus-eluting stent (EES)	847	424–1,271	±50%; uniform	[12]
Bare metal stent (BMS)	440	220–660	±50%; uniform	[12]
Myocardial infarction (MI)				
Initial STEMI, discharged alive	926	463–1,389	±50%; uniform	[12]
Initial STEMI, discharged dead	1,817	909–2,726	±50%; uniform	[12]
Repeat MI, discharged alive	2,846	1423–4,269	±50%; uniform	[12]
Repeat MI, discharged dead	3,737	1869–5,606	±50%; uniform	[12]
Stent thrombosis (STT) event				
Initial event, discharged alive	926	463–1,389	±50%; uniform	[12]
Initial event, discharged dead	1,817	909–2,726	±50%; uniform	[12]
Repeat event, discharged alive	2,846	1423–4,269	±50%; uniform	[12]
Repeat event, discharged dead	3,737	1869–5,606	±50%; uniform	[12]
Revascularization				
Coronary artery bypass graft (CABG)	16,068	8,034–24,102	±50%; uniform	[12]
Percutaneous coronary intervention (PCI) without MI	1,920	960–2880	±50%; uniform	[12]
Annual cardiovascular outpatient treatment and drug costs (excluding revascularization therapy costs)	327	164–491	±50%; uniform	Assumption^1^
Long-term annual cardiovascular treatment costs after year 5 (including revascularization therapy costs)	1,135	568–1,703	±50%; uniform	[13, 14]
Anti-platelet therapy costs after revascularization event (12 months)	240	120–360	±50%; uniform	Assumption^2^
**Life expectancy (in years)**				
5-year follow-up survivors	*		±25%; triangular	[8],[9]
**Utility scores and decrements**				
Baseline utility score			±25%; triangular	[10]
Initial STEMI event				
First year after event	-0.120	-0.090 - -0.150	±25%; triangular	[11]
Subsequent years after event	-0.085	-0.064 - -0.106	±25%; triangular	[11]
Repeat MI event				
First year after event	-0.060	-0.045 - -0.075	±25%; triangular	Assumption^3^
Subsequent years after event	-0.043	-0.032 - -0.054	±25%; triangular	Assumption^3^
STT event				
First year after event	-0.060	-0.045 - -0.075	±25%; triangular	Assumption^3^
Subsequent years after event	-0.043	-0.032 - -0.054	±25%; triangular	Assumption^3^
Revascularization (CABG or PCI)				
First year after event	-0.060	-0.045 - -0.075	±25%; triangular	Assumption^4^
Subsequent years after event	0.000		NA	Assumption^5^
Decrement due to ageing	*		±25%; triangular	[10]

EES = everolimus-eluting stent. BMS = bare metal stent. MI = myocardial infarction. STEMI = ST-segment elevation myocardial infarction. STT = stent thrombosis. CABG = coronary artery bypass graft. PCI = percutaneous coronary intervention.

* = age dependent

^1^ = assumed to be half as high as long-term annual cardiovascular treatment costs.

^2^ = assumed to cost around €20 per month for a duration of twelve months.

^3^ = assumed to affect utility only half as much as the initial STEMI event.

^4^ = assumed to have the same impact on utility as repeat MI or STT events for a year.

^5^ = no effect was assumed for subsequent years.

Clinical events and procedures which happened up to one day apart were considered as a single event, with an impact on utility equivalent to that of the more serious of the events involved. Other events were treated separately.

The final utilities were applied to the relevant time periods, and summed to compute QALYs over a life-long time horizon.

### Costs

We examined the difference in direct medical costs between the EES and BMS treatment group from a Spanish health service perspective. Drivers of resource use and resource items taken into account during the initial hospitalization period and until the end of follow-up included the number of stents, PCI and CABG procedures, as well as clinical events such as repeat MI, STT, revascularization and death. Corresponding unit costs representing 2016 prices were derived from the Spanish diagnosis-related group-based system of hospital reimbursement [12] (see Table 2). Initial hospitalization length of stay was on average 4.5 days (minimum: 0 days, maximum: 58 days) and 4.6 days (minimum: 0 days, maximum: 62 days) for patients receiving EES and BMS, respectively. The average duration of the initial PCI was 46 minutes (minimum: 9 minutes, maximum: 297 minutes) in the EES group and 46 minutes (minimum: 8 minutes, maximum: 195 minutes) in the BMS group. Initial hospitalization as well as duration of initial PCI were very similar and not significantly different between the two treatment groups based on Mann-Whitney U tests and hence, DRG-based unit costs were not corrected for such differences.

Long-term annual cardiovascular treatment costs were estimated from a published UK model of thrombolysis versus primary PCI in MI patients [13]. It was designed to incorporate the impact of repeat MIs on a summary basis, after an initial time period during which cardiovascular events were accounted for separately. The cost estimate for the UK was adapted to Spain based on current health expenditures, purchasing power parities and inflation rates. Annual cardiovascular outpatient treatment and drug costs during the follow-up period of the EXAMINATION trial, when cardiovascular events were still accounted for separately, were assumed to be half as high. Anti-platelet therapy costs up to year five were excluded from the annual cardiovascular costs and modeled separately to account for differences in revascularization rates between the EES and BMS treatment groups. It was assumed that anti-platelet therapy after a revascularization event would cost around €20 per month and would be prescribed for a duration of twelve months.

### Implementation of cost-effectiveness analysis

Actual patient-level data from the EXAMINATION trial (S1 Dataset) were employed in the cost-effectiveness analysis instead of aggregate clinical parameters or simulated patient cohorts. Other parameters from external sources, such as unit costs, life expectancy and utilities, were added to the trial dataset. A yearly discount of 3% to future costs and QALYs after the first year was used following Spanish guidelines on economic evaluations and health technologies assessments [15].

The main outcomes were total costs and QALYs for the two treatment strategies, as well as the ICER (expressed as cost per QALY gained). The ICER was compared to a willingness-to-pay threshold of €25,000 per QALY gained, which roughly corresponds to three times the gross domestic product per capita for Spain in 2016 [16] as suggested by WHO recommendations [17].

The analysis was subject to two main types of uncertainty: uncertainty over assumptions and the choice of values for parameters not covered by the trial data, as well as stochastic uncertainty due to variation in the sampled data. Parameter uncertainty results from imperfect information on analysis parameters, while stochastic uncertainty arises from the random variability in outcomes between identical patients. In order to address both types of uncertainty simultaneously and to estimate a total uncertainty range for incremental costs, we combined non-parametric bootstrapping with probabilistic sensitivity analysis [18]. The bootstrapping method randomly samples with replacement from the original data to estimate the sampling distribution for the statistic of interest. As it is a non-parametric method, no assumptions on the parametric distribution of the data need to be considered. In the probabilistic sensitivity analysis sets of input parameters are drawn by random sampling multiple times from distributions assigned to all parameters. This approach results in distributions of the outputs which can be further analyzed.

The main model run on all individual observations for 10,000 replications using bootstrapping implemented in Stata/MP 13.1 for Windows (StataCorp LP, College Station, Texas, USA). Analysis parameters subject to uncertainty were varied with each iteration. Ranges of variation and distribution types that reflect parameter uncertainty are provided in Table 2. Estimates of costs, QALYs and the ICER were obtained for each replication. The combined results of all replications were then averaged and 95% uncertainty ranges were generated with the percentile method. A 95% confidence ellipse of the incremental cost-effectiveness estimates on a cost-effectiveness plane was calculated using the ellip command of the sj4-3/gr31_1 Stata package. Model stability was evaluated for the main modelling outcomes based on subsets of the first 10 to 1,000 replications (in steps of 10 replications) and compared to the whole set of replications.

The robustness of model results was assessed via a deterministic sensitivity analysis and scenario analyses run in Stata/MP 13.1. For the sensitivity analysis, all observations from the patient-level data were included but the model additional analysis parameters subject to uncertainty were varied on a one-by-one basis, while all other parameters were fixed at their base case values. The sensitivity models were run without bootstrapping in order to describe the impact of each parameter on outcomes without the additional impact of stochastic uncertainty. In order to establish a reference point under similar conditions, all parameters were treated as fixed at their expected values, which resulted in deterministic estimates of the outcomes. The sensitivity results were then compared with those of this reference analysis to identify the most influential parameters. The models were run for 1,000 replications each.

An additional sensitivity analysis was implemented to assess the impact of stent costs on estimated patient lifetime costs and ICER. Different prices per EES were explored, ranging from €440 and €840 in steps of €50, to allow for price differences between EES and BMS of €0 up to €400. Each model was run for 1,000 replications accounting for parameter uncertainty in all parameters but without bootstrapping. The combined results of each model were averaged and 95% uncertainty intervals were generated using the percentile method.

Three different scenario analyses based on the main model were performed to assess the impact of (i) a discount rate of 0%, (ii) a discount rate of 6%, and (iii) exclusion of the patient-level data from Italy and the Netherlands. Assumptions on long-term life expectancy, health-state utility values and unit costs were drawn from published literature on Spain only, but the full EXAMINATION dataset included data from Italy and the Netherlands as well. Hence, the patient-level data from Spain only were used in scenario (iii) in order to assess the possible influence of between-country variability. All three scenario models were run for 10,000 iterations.

## Results

### Effectiveness and cost

The results of the main analysis (Table 3) indicated clinical effectiveness of EES over BMS in 89.5% of the simulations, with an incremental gain of 0.10 QALYs (95% uncertainty range: -0.06; 0.27) during the patients' lifetimes. This was attributable to longer undiscounted patient survival time predictions after receiving EES due to a statistically significant absolute reduction in the rate of all-cause mortality at the 5-year follow-up and lower clinical event rates (see Table 1).

**Table 3:** Mean cost, effectiveness and cost-effectiveness results with 95% uncertainty ranges.

Parameter	EES (n = 751)	BMS (n = 747)	Δ (EES-BMS)
**Per patient costs (in €)**			
Stent costs	1,183 (1,136; 1,232)	600 (577; 623)	583 (529; 637)
Cost of repeat MI	125 (84; 171)	90 (55; 128)	35 (-22; 92)
Cost of STT	56 (28; 87)	72 (41; 107)	-16 (-61; 29)
Cost of revascularization	318 (227; 428)	587 (426; 768)	-269 (-474; -75)
Cost of repeat CABG	85 (13; 180)	322 (167; 499)	-237 (-432; -58)
Cost of repeat PCI	233 (187; 281)	265 (216; 315)	-32 (-101; 36)
Outpatient cardiovascular treatment and drug costs up to year 5 (including anti-platelet therapy)	1,584 (1,556; 1,612)	1,567 (1,537; 1,595)	17 (-23; 57)
Total cardiovascular treatment costs over lifetime	8,123 (7,857; 8,399)	8,032 (7,725; 8,342)	91 (-315; 501)
Total costs over 5-year follow-up*	3,160 (3,024; 3,313)	2,813 (2,634; 3,009)	347 (108; 585)
Total costs over lifetime*	8,305 (8,069; 8,546)	7,874 (7,597; 8,161)	430 (58; 797)
**Survival (in years)**			
Long-term survival	10.52 (10.25; 10.79)	10.38 (10.08; 10.67)	0.14 (-0.26; 0.54)
**Effectiveness (in QALYs)**			
QALYs gained per patient over 5-year follow-up*	3.05 (3.00; 3.10)	3.00 (2.95; 3.05)	0.05 (-0.02; 0.12)
QALYs gained per patient over lifetime*	5.22 (5.11; 5.33)	5.12 (5.00; 5.24)	0.10 (-0.06; 0.26)
**Cost-effectiveness (in € per QALY gained)**			
ICER over 5-year follow-up*			6,890 (-30,745; 44,155)
ICER over lifetime*			3,948 (-17,168; 24,580)

EES = everolimus-eluting stent. BMS = bare metal stent. MI = myocardial infarction. STT = stent thrombosis. CABG = coronary artery bypass graft. PCI = percutaneous coronary intervention. QALY = quality-adjusted life years. ICER = incremental cost-effectiveness ratio.

**Δ** = difference.

* = discounted by 3%.

Patient lifetime costs were €430 higher in the EES than in the BMS strategy, with a 95% uncertainty range between €58 and €797 indicating statistical significance. This was mainly driven by higher stent costs. Only in 1.4% of model runs, the total discounted costs of the BMS strategy exceeded the costs of the EES strategy. Significant cost savings in the EES strategy were observed for revascularizations (especially CABG costs), which was related to lower revascularization event rates.

The model appeared to be stable after the first 400–500 replications when comparing the results of the first 10 to 1,000 replications (results not presented). Costs and QALYs at 1,000 replications (EES: €8,302 (95% uncertainty range: €8,051-€8,543), 5.22 (5.11–5.33); BMS: €7,876 (€7,600-€8,151), 5.12 (4.99–5.24)) were almost identical with the full set of 10,000 replications for both treatment strategies indicating high accuracy of the estimates at 10,000 replications.

### Cost-effectiveness

**Fig 1:**
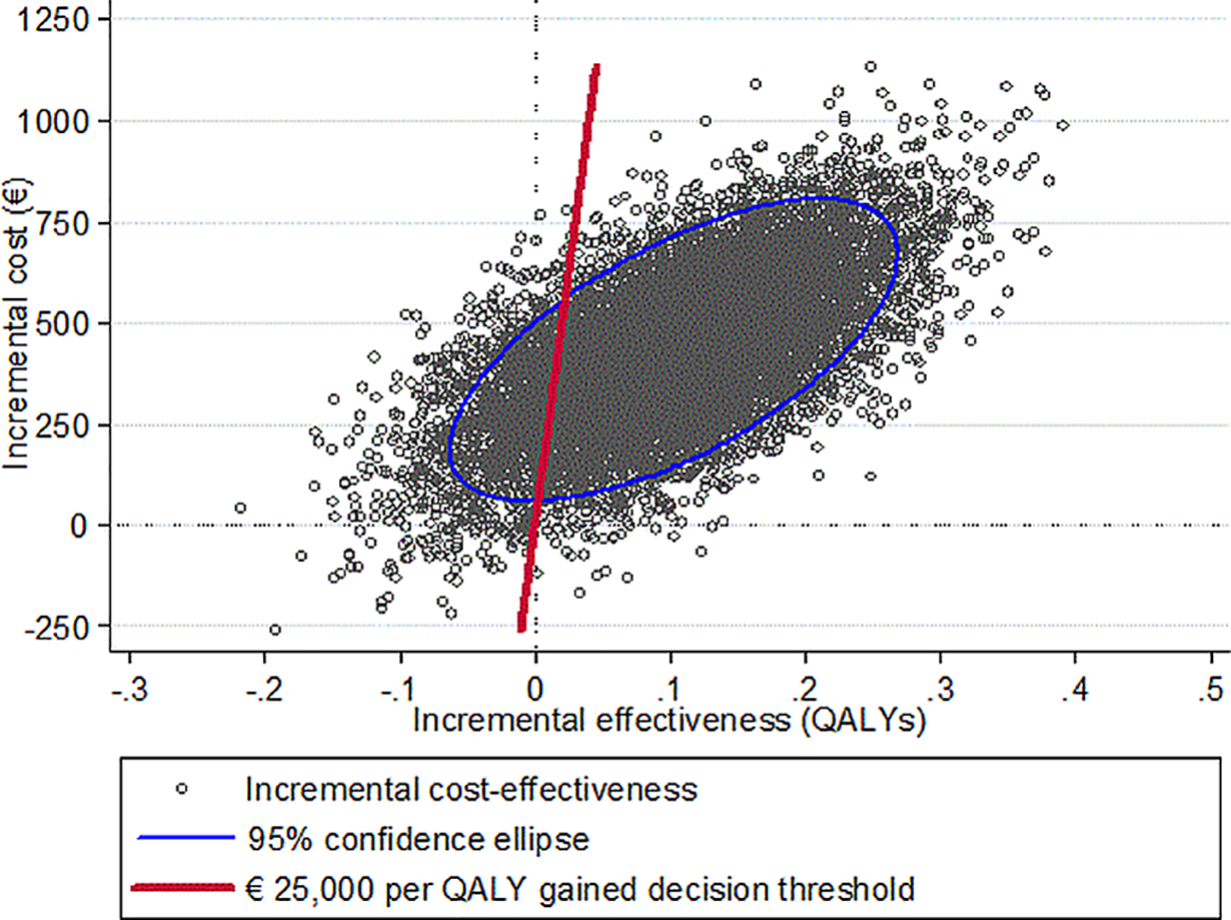
Cost effectiveness plane. QALY = quality-adjusted life-year.

The joint distribution of incremental costs and effectiveness is shown in Fig 1. In 0.3% of the simulations the EES strategy was dominant (clinically effective and cost saving; fourth quadrant of Fig 1) while BMS was dominant in 9.9% of the cases (second quadrant).

**Fig 2:**
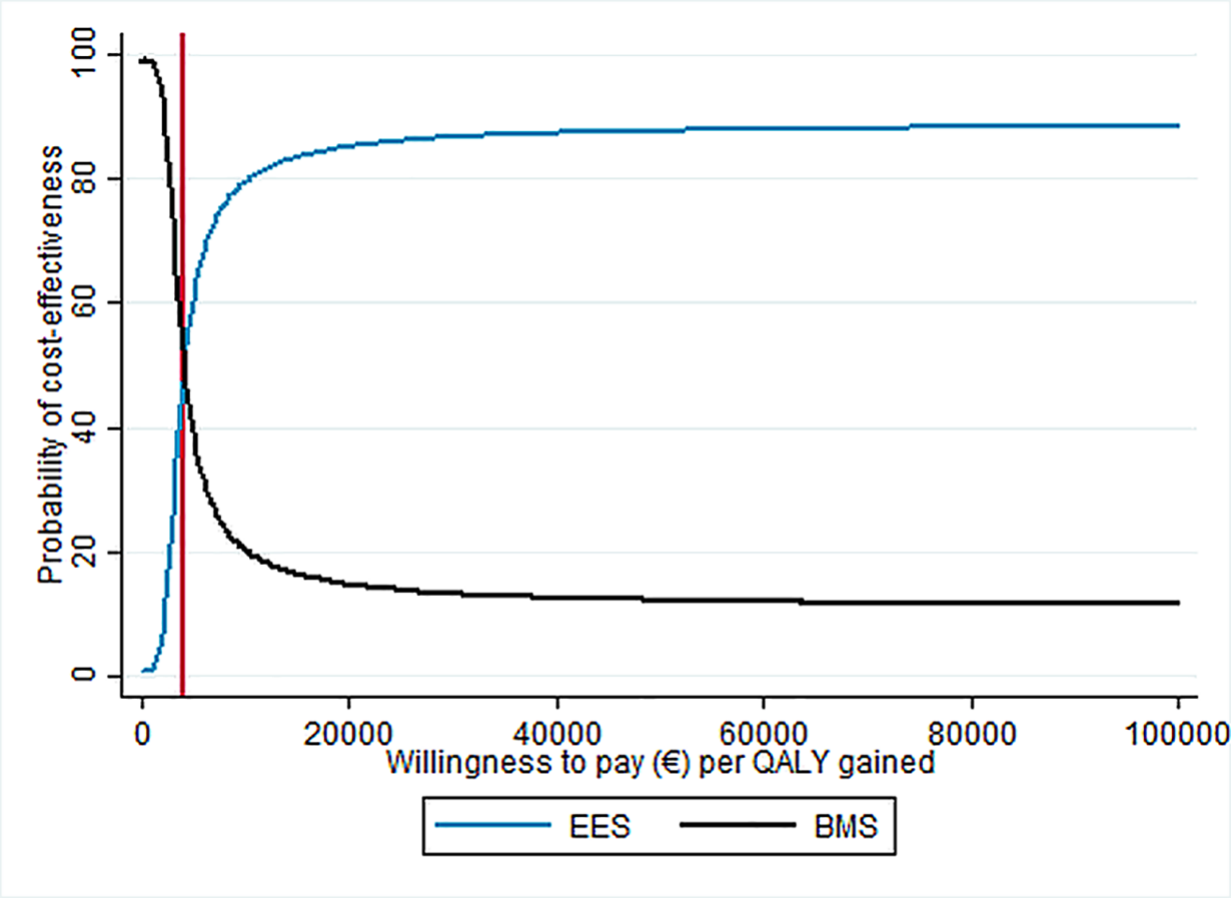
Cost-effectiveness acceptability curve. BMS = bare-metal stent. EES = everolimus-eluting stent. QALY = quality-adjusted life-year.

The average ICER over all simulations was €3,948 (median: €3,594) per QALY gained and was below a willingness-to-pay threshold of €25,000 per QALY gained in 86.9% of the simulations, indicating cost-effectiveness of EES over BMS. The probability of cost-effectiveness based on different levels of willingness-to-pay is presented in Fig 2. For a willingness-to-pay of at least €4,169 per QALY gained, EES appeared to be more likely to be cost-effective than BMS.

### Sensitivity analysis

**Fig 3:**
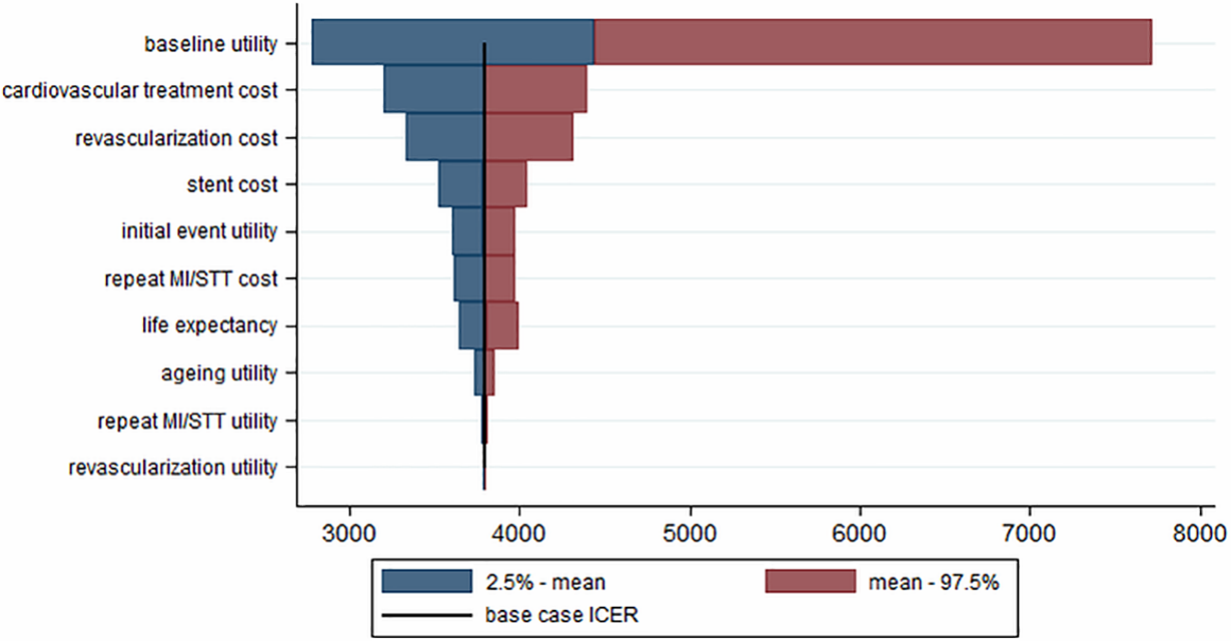
Tornado diagram showing the impact of uncertainty on the outcome of the modelling parameters. ICER = incremental cost-effectiveness ratio. MI = myocardial infarction. STT = stent thrombosis.

The main analysis addressed the uncertainty due to random variability in the trial data and the uncertainty over the choice of analysis parameter values from sources external to the trial, simultaneously. The uncertainty introduced by each of the external parameters (while considering the other parameters as fixed at their base case value), applying a model without non-parametric bootstrapping, is presented in Fig 3. The ICER point estimate resulting from the reference analysis (with all parameters fixed and all trial observations used) was €3,797 per QALY gained. The corresponding incremental effectiveness and cost results were similar to the estimates of the main analysis (0.11 vs. 0.10 QALYs gained; €426 vs. €430). Irrespective of the parameters considered, the sensitivity analyses showed that EES were more costly than BMS but clinically more effective. Parameters introducing the largest uncertainty, indicated by the widest intervals for the ICER, were the baseline utility scores, cardiovascular treatment and revascularization costs.

**Fig 4:**
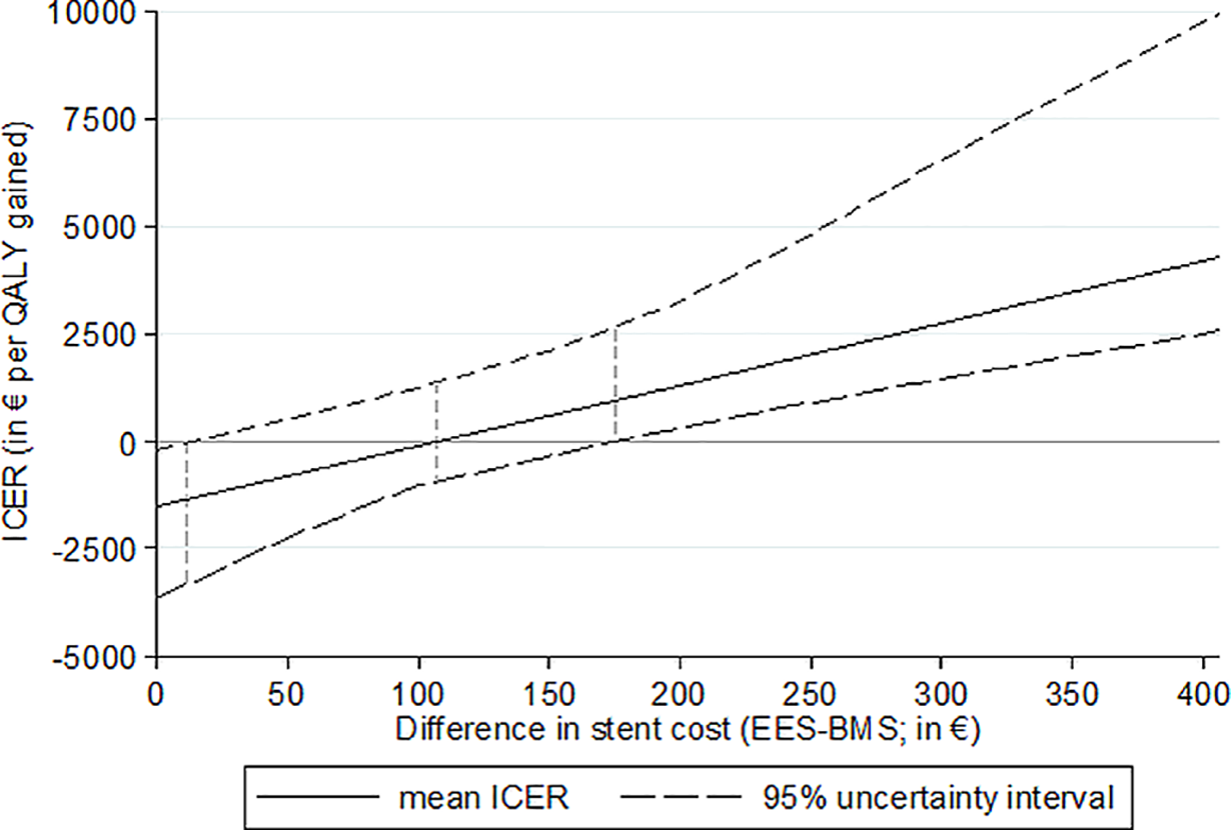
Graph showing the impact of the difference in stent cost on the ICER. BMS = bare-metal stent. EES = everolimus-eluting stent. ICER = incremental cost-effectiveness ratio. QALY = quality-adjusted life-year.

The impact of varying costs per EES on the ICER is shown in Fig 4. Costs per EES of €615 (corresponding to a cost difference of €175 vs. BMS) and above would result in higher patient lifetime costs of the EES strategy based on the 95% uncertainty interval of the ICER. At a cost difference of €110 (with a 95% uncertainty interval ranging from €12 to €174), the average ICER would equal zero and patients' lifetime costs would be the same in the EES and BMS strategy. If the cost per EES dropped below €452 (corresponding to a cost difference of less than €12), the EES strategy would dominate the BMS strategy.

### Scenario analyses

The first scenario analysis with a discount rate of 0% (i) generated incremental costs of €420, similar as in the main analysis, which indicated that a large proportion of the cost difference was attributable to the first few years after the original STEMI event. The incremental effectiveness of 0.12 QALYs was increased as compared to the main analysis. The ICER was €2,142 per QALY gained (95% uncertainty range: €-15,335 - €19,354; median: €2,272). In 79% of the replications, the willingness-to-pay threshold of €25,000 per QALY gained was met.

With a discount rate of 6% (ii), the incremental costs (€422) remained again similar but the incremental effectiveness was reduced to 0.08 QALYs. The estimated ICER was €5,986 per QALY gained (95% uncertainty range: €-18,169 - €37,144; median: €4,995). The willingness-to-pay threshold of €25,000 per QALY gained was met in 88% of the replications.

The scenario analysis based on the Spanish EXAMINATION patients only (iii) contained about 76% (1,138 of 1,498) of the patient-level observations. The predicted incremental costs increased by 24% compared to the main analysis (€533 vs. €430), while the incremental effectiveness remained the same (0.10 QALYs). The increased costs were mainly due to larger differences between the simulated repeat MI, revascularization and cardiovascular treatment costs. The average ICER was estimated to be €4,728 (95% uncertainty range: €-28,312 - €29,064; median: €4,193) per QALY gained. About 83% of the results were below a willingness-to-pay threshold of €25,000 per QALY gained.

## Discussion

### Principal findings

Our analysis based on the 5-year follow-up of the EXAMINATION trial indicated that the use of EES in STEMI patients is clinically effective as well as cost-effective compared to the use of BMS, despite higher total costs. It is likely to be preferable from a Spanish health service perspective, with an ICER estimate of €3,948 per QALY gained. Cost-effectiveness estimates remained stable in sensitivity analyses and were highly consistent in scenario analyses.

### Comparison with other economic evaluations

We performed the first incremental cost-effectiveness analysis of the EXAMINATION trial, and, as far as we are aware, the first cost-effectiveness analysis of EES versus BMS with a lifelong time horizon and with a focus on STEMI patients. Another recent economic evaluation, by Ferko et al., addressed the cost-effectiveness of cobalt chromium EES *versus* BMS in PCI based on a Markov state transition model including patient-level data from meta-analysis of randomized controlled trials [19]. The authors found EES to be economically dominant over BMS in contemporary US clinical practice. Cost savings of US$236 attributed to reductions in target vessel revascularizations and MI rates were estimated. In contrast to our analysis, Ferko et al. focused on a time frame of two years, hence underestimating the cost of outpatient cardiovascular treatment over a lifelong time horizon but also quality-adjusted survival time. While we assumed no difference in the long-term survival of 5-year survivors, more patients enter the post-5-year period in the EES strategy. This leads to a difference in total survival time, and thus difference in time requiring treatment of cardiovascular disease, between the strategies. Applegate et al. [20] also reported that EES reduced target lesion revascularization rates and were cost-effective over BMS, across strata defined by stented vessel length and vessel diameter, based on propensity score matching.

Very recently, Poder et al. [21] presented a cost-benefit analysis of second generation drug-eluting stents versus BMS based on systematic review of meta-analyses with unit costs of the Quebec health care system and adopting a 2-year perspective. They reported that second generation drug-eluting stents, especially EES, lower target-vessel revascularization and MI rates and cause fewer deaths and in-stent thrombosis than BMS. Their results indicated savings of $479 per patients if all eligible patients would receive second generation drug-eluting stents. Even higher savings could be observed if the patient's risk of reintervention is high.

### Strengths and limitations

The EXAMINATION trial was a large multicenter, multinational, prospective, randomized, two-arm, single-blind, controlled trial allowing for a high level of internal validity and predominantly conducted in Spain. The use of local data on life-expectancy, utility scores representing health-related quality of life, and unit costs allowed us to reflect the Spanish health service environment. As about one quarter of the EXAMINATION data were from patients enrolled in Italy and the Netherlands, we performed an additional scenario analysis based on data from Spain only. This resulted in consistent incremental effectiveness and somewhat higher incremental costs. Nevertheless, the results still indicated the use of EES to be cost-effective.

When the EXAMINATION trial was designed the anticipated start of the study was in late 2008. By that time, it was unclear whether drug-eluting stents (DES) would be superior to BMS in a pro-thrombotic scenario like STEMI, given the evidence at that time of a higher thrombosis rate of DES *versus* BMS. On top of this, BMS were also less expensive than DES so that STEMI was a niche for BMS use. The EXAMINATION trial demonstrated that DES were superior to BMS in terms of major adverse clinical events, showing also a lower rate of thrombosis. Nevertheless, in some non-European Union countries, use of BMS in STEMI is still defended due to its lower cost compared to DES. For this reason, our first aim was to explore whether the higher efficacy of DES counterbalances their higher cost compared to BMS. We believe that our findings are still of interest even though DES became widely used in many settings as such large studies with long-term patient-level clinical data are rare and given the continued use of BMS at least in resource-restricted settings. The presented results might also be used in network meta-analyses which draw inference from different studies that share certain treatments in order to compare treatments of interest for which there is no head-to-head comparison available.

The EXAMINATION trial had broad inclusion and few exclusion criteria to ensure an all-comers population of adult STEMI patients which is representative of routine clinical practice [4]. Specifically, after signing the informed consent, patients could be included if suffering from STEMI up to 48 hours after the onset of symptoms requiring emergent PCI. In case the patient was unable to provide written informed consent, a legally acceptable representative was entitled to sign the form. The only anatomic exclusion criterion was a vessel size larger than 4.0 mm or smaller than 2.25 mm. The population included in the EXAMINATION trial represented 70% of the patients screened. As the population is assumed to almost represent daily clinical practice, the effect on the outcomes of our study is considered to be small given that we also considered patient-level variation within our models.

Given the patient-level clinical data of the EXAMINATION trial, we were able to account for uncertainty in input parameters external to the trial data, and patient-level variation reflected in the trial data, simultaneously using non-parametric bootstrapping coupled with a probabilistic sensitivity analysis. Bootstrapping methods are still commonly used in cost-effectiveness analyses in health technology assessment [22, 23]. An alternative approach are Bayesian methods. Bayesian cost-effectiveness analyses provide similar results compared to non-parametric bootstrapping when vague priors are applied but the results could be enhanced if informative priors are considered [24]. When patient-level data are not available, assumptions on the distribution of clinical events across patients have to be made based on summary estimates such as average risk, relative risk or hazard ratio. However, errors may be introduced as such estimates cannot account for the possibility of uneven event occurrence across patients. Such problems are automatically dealt with in an analysis based on patient-level data.

All input parameters external to the trial data (e.g. unit costs) were varied widely in sensitivity analysis. The effect on the main outcomes was low. The upper bounds of the ICER confidence intervals remained below €8,000 per QALY gained. Even for parameters for which we needed to rely on relatively uncertain assumptions, such as those representing cardiovascular treatment costs or utility scores for clinical events after the initial STEMI event, the amount of uncertainty introduced into the analysis was small and the EES strategy remained cost-effective over BMS.

We made an assumption that clinical events and procedures happening up to one day apart were to be considered as a single event in terms of their impact on utility. We applied, in such cases, the effect of the more serious event or procedure. This may have introduced an element of error in the estimation of utility decrements, and hence, QALYs gained.

For the analysis, we focused on the first occurrence of each type of clinical event that was recorded for each patient. Several revascularization, repeat MI or STT events may have occurred in some patients, implying an underestimation of event rates. This may have introduced an error to our cost and effectiveness estimates, such that effectiveness may have been overestimated and costs underestimated. However, the occurrence of repeat events is rather low and was lower in the EES group [5]. Hence, the effect on the ICER might be marginal and potentially favor the EES strategy.

The model assumed that long-term survival after the 5-year follow-up period was the same between the two treatment groups. However, differential survival rates might occur even after such an extended follow-up period. If the survival advantage was sustained in the EES group, which might be a plausible assumption, incremental effectiveness would increase, as well as incremental costs due to larger long-term cardiovascular treatment costs. However, the survival advantage could also be used up over time, resulting in a lower incremental effectiveness of EES and hence the advantage of EES over BMS could become less clear-cut.

Main drivers of the €430 higher lifetime cost in the EES strategy were stent, cardiovascular treatment and repeat MI costs. During the five years of follow-up, the difference in the outpatient cardiovascular treatment and drug costs, including antiplatelet therapy costs, was only €17. Hence, most of the difference in treatment costs was accumulated after the follow-up period and was substantially driven by higher survival of EES patients and the resulting prolonged period on cardiovascular treatment. In contrast, the costs for STT and revascularization events were lower in the EES than in the BMS group due to higher clinical event rates of patients receiving BMS.

We consider the analysis to remain reasonably accurate considering current clinical practice, which can be different from guidelines. In any case, as cost estimation was performed in the same way in the two groups compared, we would not expect any related differences to strongly influence our results.

Our model currently assumes that clopidogrel is used as part of the lifetime outpatient cardiovascular treatment. If we assume that ticagrelor, which is a more expensive alternative drug, would be used instead, the incremental costs would increase due to enlarged treatment cost. Hence, cost-effectiveness of the EES strategy would decrease. However, even at three times higher costs of ticagrelor in comparison with clopidogrel and assuming that there is no effect on survival as well as utilities, the resulting ICER would remain below a willingness-to-pay threshold of €25,000 per QALY gained and the EES strategy would remain preferable over BMS.

While the World Health Organisation's (WHO's) Choosing Interventions that are Cost–Effective project is recommending a cost-effectiveness threshold of three times the national gross domestic product per capita [17], there is no formally accepted cost-effectiveness threshold for Spain. Only in the UK, an official threshold of £20,000–30,000 per QALY gained [25] exists. In studies for Switzerland, thresholds of up to CHF 100,000 per QALY gained are frequently considered and in studies for the USA, US$ 100,000 per QALY gained or even higher are common reference points. For this analysis, we tentatively compared results against a threshold of €25,000 per QALY gained, consistent with the WHO recommendation [17]. On this basis, EES can be reasonably considered as cost-effective in comparison with BMS.

While clinical outcomes may be comparable across different healthcare systems, other elements such as unit costs, utilities, long-term survival, healthcare costs, policies governing stent use and geographic context are often not. Given the focus on Spain, our results may not allow to draw direct inferences for other countries. However, since the average ICER was well below typically used cost-effectiveness thresholds, it may be reasonable to expect that the EES strategy is also cost-effective in comparison with the BMS strategy in other European countries with a similar socio-economic structure. Specific assessments for additional jurisdictions would imply to re-run the analyses with local estimates for the above-listed non-trial based elements, assuming that the clinical outcomes would be similar to those obtained from the EXAMINATION trial.

### Further research

We focused on comparing EES against BMS from a Spanish health service perspective. Drawing firm conclusions for different settings would require to obtain patient-level clinical data from a variety of countries. Furthermore, it would be important to obtain even longer-term data to assess whether the survival advantage of EES patients and reduction in clinical event rates can be sustained.

## Conclusion

In STEMI patients requiring emergency PCI, the use of EES instead of BMS appeared to be a cost-effective therapy from a Spanish health service perspective due to its clinical benefits and incremental effectiveness despite of higher total costs. Predicted ICERs were below generally accepted threshold values which was confirmed by sensitivity analyses.

## Supporting information

S1 Dataset Minimal anonymized data set to replicate study findings. (XLSX)
